# Distinct eye movement patterns to complex scenes in Alzheimer’s disease and Lewy body disease

**DOI:** 10.3389/fnins.2024.1333894

**Published:** 2024-04-05

**Authors:** Yasunori Yamada, Kaoru Shinkawa, Masatomo Kobayashi, Miyuki Nemoto, Miho Ota, Kiyotaka Nemoto, Tetsuaki Arai

**Affiliations:** ^1^Digital Health, IBM Research, Tokyo, Japan; ^2^Department of Psychiatry, Division of Clinical Medicine, Institute of Medicine, University of Tsukuba, Tsukuba, Ibaraki, Japan

**Keywords:** saliency model, mild cognitive impairment, dementia, free viewing, differential diagnosis, brain atrophy, Alzheimer’s disease, dementia with Lewy bodies

## Abstract

**Background:**

Alzheimer’s disease (AD) and Lewy body disease (LBD), the two most common causes of neurodegenerative dementia with similar clinical manifestations, both show impaired visual attention and altered eye movements. However, prior studies have used structured tasks or restricted stimuli, limiting the insights into how eye movements alter and differ between AD and LBD in daily life.

**Objective:**

We aimed to comprehensively characterize eye movements of AD and LBD patients on naturalistic complex scenes with broad categories of objects, which would provide a context closer to real-world free viewing, and to identify disease-specific patterns of altered eye movements.

**Methods:**

We collected spontaneous viewing behaviors to 200 naturalistic complex scenes from patients with AD or LBD at the prodromal or dementia stage, as well as matched control participants. We then investigated eye movement patterns using a computational visual attention model with high-level image features of object properties and semantic information.

**Results:**

Compared with matched controls, we identified two disease-specific altered patterns of eye movements: diminished visual exploration, which differentially correlates with cognitive impairment in AD and with motor impairment in LBD; and reduced gaze allocation to objects, attributed to a weaker attention bias toward high-level image features in AD and attributed to a greater image-center bias in LBD.

**Conclusion:**

Our findings may help differentiate AD and LBD patients and comprehend their real-world visual behaviors to mitigate the widespread impact of impaired visual attention on daily activities.

## 1 Introduction

Visual attention and eye movements involve a widely distributed network of brain regions (Corbetta and Shulman, 2002; Miller and Buschman, 2013), and altered eye movements can reflect cognitive and motor impairments in neurodegenerative disorders (Anderson and MacAskill, 2013; Leigh and Zee, 2015). Quantifying complex patterns of these alterations may help differentiate disorders as behavioral biomarkers (Anderson and MacAskill, 2013; Tseng et al., 2013; Itti, 2015) and enhance our comprehension of disease-associated alterations in patients’ real-world visual behaviors (Molitor et al., 2015; Ramzaoui et al., 2018). Alzheimer’s disease (AD) and dementia with Lewy bodies (DLB), one form of the Lewy body diseases (LBDs), are the two most common subtypes of late-onset neurodegenerative dementias (Walker et al., 2015; Crous-Bou et al., 2017; McKeith et al., 2017) and exhibit comparable clinical manifestations, posing challenges in their differential diagnosis (Walker et al., 2015; McKeith et al., 2017; Chatterjee et al., 2021). Patients with AD and DLB often exhibit impaired visual attention (Collerton et al., 2003; McGuinness et al., 2010; Revie et al., 2019) and altered eye movements (Anderson and MacAskill, 2013; Das et al., 2022). Studies on single-feature and feature-conjunction visual search tasks suggest impaired top-down attentional guidance in DLB compared with AD, with bottom-up attentional guidance preserved (Cormack et al., 2004; Landy et al., 2015; Ramzaoui et al., 2018). In particular, DLB patients tend to have larger alterations of eye movements compared with AD patients (Cormack et al., 2004; Mosimann et al., 2005), corroborated by greater impairments in attention-relevant neuropsychological examinations (Collerton et al., 2003; Wood et al., 2013; Revie et al., 2019) and prominent changes in neural networks related to visual attention (Taylor et al., 2013; Landy et al., 2015). However, all these prior studies have used artificial stimuli like simple arrays of geometrical shapes (e.g., dots and squares), which may not represent real-world viewing behaviors. Consequently, research utilizing naturalistic complex stimuli representing those that individuals may encounter in everyday life is increasingly gaining importance owing to its ecological validity (Wang et al., 2015; Ramzaoui et al., 2018).

Several studies on complex scene viewing in AD patients, but not DLB patients, have reported two remarkable alterations in top-down attentional guidance and visual exploration. Studies using simple stimuli or complex scenes (photographs of streets) have reported reduced task-appropriate modulations of eye movements (Redel et al., 2012; Shakespeare et al., 2015) and fewer fixations on task-related informative locations (Mosimann et al., 2004; Tokushige et al., 2023), suggesting impaired top-down attentional guidance in AD patients (Redel et al., 2012; Shakespeare et al., 2015; Ramzaoui et al., 2018). Other studies have investigated free viewing of complex scenes with an incongruous element (Daffner et al., 1992) or non-social scenes (environments without animate objects like humans or animals) (Coco et al., 2021), reporting diminished visual exploration with fewer fixated regions in AD or mild cognitive impairment (MCI), possibly related to apathy (a lack of motivation and interest) or cognitive impairments such as slowing processing speed and difficulties in disengaging and shifting attention (Daffner et al., 1992, 1999; Molitor et al., 2015; Seligman and Giovannetti, 2015). Although DLB patients have not been studied in regards to visual exploration patterns, such patients might show a similar reduction in visual exploration since apathy and cognitive impairments, particularly in attention, have also been observed in DLB patients (Collerton et al., 2003; McGuinness et al., 2010; Bjoerke-Bertheussen et al., 2012; Revie et al., 2019). However, the prior studies still used restricted stimulus sets in terms of their numbers (typically less than ten) and categories (only non-social scenes). Therefore, a systematic characterization of altered eye movements on complex scenes containing broader categories of objects that could influence attention in AD and DLB patients has yet to be determined. Furthermore, although top-down attentional guidance can be influenced by both task demands and high-level image features such as faces, texts, and emotions even during free viewing (Mannan et al., 2009; Borji et al., 2012, 2013; Wang et al., 2015), the extent to which attention bias toward such high-level image features differs in AD or DLB patients remains uninvestigated.

Here, we aimed to assess spontaneous gaze allocation in patients with AD and LBD at the MCI and dementia stages to naturalistic complex scenes with broad categories of objects, assuming that it would further reflect real-world viewing behaviors. The stimulus set consisted of 200 different scene images labeled with high-level image features of object-based semantic attributes, such as object properties (e.g., shape) and semantic information (e.g., faces), annotated on a total of 1,533 manually-segmented objects. This enabled us to investigate the differences in attention bias toward these high-level features using a data-driven approach with a computational visual attention model (Xu et al., 2014; Wang et al., 2015). By comparing with matched controls, we tested the following hypotheses: (i) both AD and LBD patients would have diminished visual exploration and weaker attention bias to high-level image features, and (ii) these reductions would be larger in LBD than in AD. Furthermore, we aimed to explore the associations of these alterations in eye movements with disease severity and brain structural changes, as well as the feasibility of using gaze allocation patterns to differentiate AD and LBD.

## 2 Materials and methods

### 2.1 Study participants

We recruited community-dwelling older adults in Ibaraki, Japan, using consecutive sampling. The participants comprised outpatients from the Department of Psychiatry at the University of Tsukuba Hospital, the spouses of the patients, and other participants recruited via local agencies or community advertisements. All participants underwent cognitive, physical, and clinical assessments as listed in Table 1.

**TABLE 1:** Participant characteristics.

	CU (*n* = 37)	AD (*n* = 49)	LBD (*n* = 20)	*P*-value
Age, years	71.4 ± 4.9 (62, 80)	73.4 ± 5.8 (54, 86)	74.2 ± 5.3 (61, 81)	0.117
Sex, female, *n* (%)	23 (62.2%)	18 (36.7%)	10 (50.0%)	0.064
Education, years	13.2 ± 1.9 (9, 18)	13.2 ± 2.9 (9, 17)	12.4 ± 2.6 (9, 17)	0.435
Disease stage, MCI, *n* (%)		31 (63.3%)	13 (65.0%)	1.000
Antipsychotic medication, intake, *n* (%)*	0 (0.0%)	2 (4.1%)	5 (25.0%)	**< 0.001** ^b,c^
Gate speed, m/s^†^	1.38 ± 0.17 (1.03, 1.74)	1.21 ± 0.21 (0.74, 1.64)	1.26 ± 0.22 (0.99, 1.67)	**0.002** ^a^
Geriatric Depression Scale^‡^	3.1 ± 2.9 (0, 10)	3.4 ± 3.1 (0, 13)	4.1 ± 4.4 (0, 14)	0.601
Mini-Mental State Examination	28.1 ± 1.5 (24, 30)	24.1 ± 3.9 (15, 30)	26.3 ± 4.1 (15, 30)	**< 0.001** ^a,c^
Logical memory–immediate	11.6 ± 3.9 (6, 22)	4.8 ± 3.3 (0, 12)	7.5 ± 4.0 (0, 14)	**< 0.001** ^a,b,c^
Logical memory–delayed	9.7 ± 3.2 (5, 17)	2.3 ± 2.7 (0, 8)	5.8 ± 4.1 (0, 12)	**< 0.001** ^a,b,c^
Frontal Assessment Battery	14.2 ± 2.2 (8, 17)	11.6 ± 3.3 (4, 18)	10.7 ± 4.4 (4, 17)	**< 0.001** ^a,b^
Trail Making Test part A^‡^	34.9 ± 12.1 (18, 84)	52.3 ± 42.5 (20, 300)	68.5 ± 61.0 (21, 300)	**0.010** ^b^
Trail Making Test part B*	83.1 ± 25.8 (35, 146)	161.9 ± 90.8 (65, 300)	185.7 ± 93.7 (68, 300)	**< 0.001** ^a,b^
Clock Drawing Test	6.7 ± 0.9 (2, 7)	6.1 ± 1.6 (1, 7)	6.5 ± 1.2 (2, 7)	0.073
Clinical Dementia Rating	0.0 ± 0.0 (0, 0)	0.6 ± 0.2 (0.5, 1)	0.6 ± 0.4 (0, 2)	**< 0.001** ^a,b^
Clinical Dementia Rating–Sum of Boxes	0.0 ± 0.1 (0, 0.5)	2.2 ± 2.0 (0.5, 6)	2.8 ± 2.7 (0, 10)	**< 0.001** ^a,b^

Values are displayed as mean ± SD (range) and were examined by using one-way analysis of variance except for categorical variables, which are displayed as *n* (%) and were examined by using chi-square test. Significant differences between individual diagnostic groups (Tukey-Kramer or chi-square test as appropriate, *P* < 0.05) were marked in bold with ^a^, ^b^, or ^c^ (^a^significant between CU vs. AD; ^b^significant between CU vs. LBD; ^c^significant between AD vs. LBD). CU, cognitively unimpaired; AD, Alzheimer’s disease; LBD, Lewy body disease; MCI, mild cognitive impairment; SD, standard deviation. *Data missing for 3 participants,

^†^Data missing for 16 participants. ^‡^Data missing for 1 participant.

The participants were categorized into three clinical diagnostic groups: AD, LBD, and cognitively unimpaired (CU). Participants in the AD group met the National Institute on Aging and Alzheimer’s Association core clinical criteria for probable AD dementia (McKhann et al., 2011) or MCI (Albert et al., 2011), as well as the AD Neuroimaging Initiative criteria for AD or MCI (Petersen et al., 2010). Those in the LBD group fulfilled McKeith et al.’s 2017, 2020 clinical diagnostic criteria for probable/possible DLB or MCI with Lewy bodies. Consequently, patients in the AD and LBD groups included those with MCI (MCI-AD and MCI-LB, respectively), and ranged from MCI to moderate dementia (Morris, 1993). The CU participants were matched for age, sex, and years of education, and did not meet any of the aforementioned criteria. Participants were excluded if they had diagnoses of other types of dementia (e.g., frontotemporal dementia, vascular dementia, and Parkinson’s disease dementia) or mental illness (e.g., major depression, bipolar disorder, and schizophrenia) at the time of the experiment, evidence of stroke, or other serious diseases or disabilities that would interfere with the eye movement data collection. All diagnoses were confirmed by three psychiatrists (authors TA, KN, and MO), who are dementia specialists and were blind to the results of the eye movement analysis, in terms of cognitive and clinical measures as well as clinical records.

A power analysis was conducted to determine the appropriate sample size for comparing the three groups using Cohen’s *f* effect size measure. On the basis of a previous study (Mosimann et al., 2005) that analyzed eye movement characteristics for AD, DLB, and controls and yielded an *f* of 0.52 to 1.00, we used *f* = 0.5 as the target effect size for between-group differences. With a significance level of 0.05 and statistical power of 0.8, the sample size of 14 for each group was considered to be sufficient to detect meaningful differences between the groups.

The study protocol was approved by the Ethics Committee, University of Tsukuba Hospital (H29-065), and was in accordance with the Declaration of Helsinki. All participants gave written informed consent to participate in the study.

### 2.2 Assessments

Cognitive assessments included the Mini-Mental State Examination (MMSE), immediate and delayed recall of the logical memory–story A of the Wechsler Memory Scale–Revised, the Frontal Assessment Battery, Trail Making Test part A and part B, and the Clock Drawing Test, all of which were conducted by experienced neuropsychologists. Clinical assessments included the Clinical Dementia Rating and Geriatric Depression Scale. Patients in the LBD group were also administered the Unified Parkinson’s Disease Rating Scale (UPDRS). For the UPDRS, we used the five-item version (Thomas et al., 2017; Thomas, 2022), which has been validated for the assessment of parkinsonism in dementia (Ballard et al., 1997) and MCI (Fernando et al., 2024), and recommended for use due to its robustness against the severity of cognitive impairment (Ballard et al., 1997; Thomas et al., 2017). The physical assessment was a nine-meter walk test, where the participants were instructed to walk nine meters at their usual pace in an unobstructed space. Gait speed was measured using an optical motion capture system (OptiTrack Flex13; NaturalPoint, Inc., Corvallis, OR, USA sample rate of 120 Hz), and the first and last two meters were excluded from analyses to discard the increase and decrease speed effect. Detailed equipment and procedural information were previously outlined (Yamada et al., 2021). We used the average gait speed across three trials as the physical assessment score for each participant.

To assess structural brain changes, we used a 1.5-T Avanto scanner (Siemens Medical System, Inc., Erlangen, Germany) with a three-dimensional magnetization-prepared rapid gradient-echo sequence yielding 160 contiguous T1-weighted slices with a 1.2-mm thickness in the sagittal plane. The imaging parameters were repetition time = 2,400 ms, echo time = 3.52 ms, flip angle = 8°, field of view = 240 mm, and matrix size = 192 × 192 pixels. The voxel size was 1.25 × 1.25 × 1.25 mm^3^. Regions of interest (ROIs) were selected on the basis of previous studies on AD (Dickerson et al., 2009; Schwarz et al., 2016) since structural imaging studies on DLB yield mixed results but report similar patterns of cortical thinning to AD (Watson et al., 2015; Ye et al., 2020). Specifically, we analyzed the following ten brain ROIs: hippocampus volume and cortical thickness of the middle temporal, inferior temporal, inferior parietal, superior parietal, supramarginal, superior frontal, entorhinal, fusiform, and precuneus regions. The magnetic resonance images were reconstructed using FreeSurfer software version 7.3.2 (Fischl, 2012) running on Ubuntu 20.04 based Lin4Neuro (Nemoto et al., 2011). The surface-based pipeline consisted of several stages, including removal of non-brain tissue, segmentation of the subcortical white matter and deep gray matter volumetric structures, intensity normalization, tessellation of the gray matter-white matter boundary, surface deformation following intensity gradients to optimally place the gray/white and gray/cerebrospinal fluid borders at the location, registration to a spherical atlas, and parcellation of the cerebral cortex into units on the basis of gyral and sulcal structures. The hippocampus volume was calculated using the Aseg atlas (Fischl et al., 2002) and normalized to intracranial volume, while the vertex-wise mean cortical thickness values were calculated for each cortical ROI using the Desikan-Killiany-Tourville atlas (Klein and Tourville, 2012).

### 2.3 Eye-tracking data collection

**FIGURE 1:**
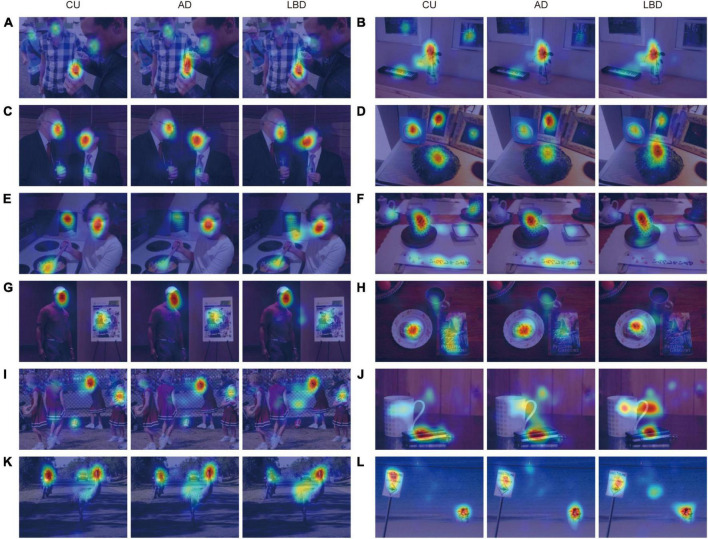
Examples of complex scene images with gaze densities derived from the CU, AD, and LBD groups. Results show that AD and LBD patients focused on fewer locations **(A–D)**, had different attention biases toward high-level semantic features **(E–H)**, and looked more at the image center **(I–L)**, compared with CU participants. The images of visual stimuli were taken from the Object and Semantic Images and Eye-tracking dataset (Xu et al., 2014).

The participants were presented with a total of 200 naturalistic complex scene images for three seconds each in two sessions (100 images for each session; see Figure 1 and Supplementary Figure 1 for examples). The stimulus duration (i.e., three seconds) was determined on the basis of previous studies investigating how neurological disorders affect eye movements and visual saliency in free-viewing conditions (Tseng et al., 2013; Wang et al., 2015). Each session was thus five minutes (three seconds × 100 images), and participants rested between the sessions. The participants were instructed to simply look at the images throughout a session. The images were randomly selected from the publicly-available Object and Semantic Images and Eye-tracking dataset (Xu et al., 2014). Briefly, the dataset comprises 700 different scenes representing a range of social and non-social situations typical of daily life. Each scene includes multiple dominant objects rather than a central one (median number of objects was 7 for the images we used; for the distribution, see Supplementary Figure 2). Each image was manually segmented into a collection of objects and was quantified in accordance with both low-level image features of pixel-level attributes (color, intensity, and orientation) and high-level image features of object-based semantic attributes that represent object properties (complexity, convexity, solidity, and eccentricity) and semantic information (face, emotion, touched, gazed, motion, sound, smell, taste, touch, text, watchability, and operability) annotated on each segmented object. Such hand-labeled stimuli have demonstrated advantages in investigating the relative contribution of multiple feature types to visual attention (Xu et al., 2014; Wang et al., 2015). The images were shown on a 20-inch computer screen (resolution = 1600 × 1200 pixels), resulting in 28.5° × 21.6° visual angles as participants were seated ∼80 cm away from the screen.

Eye-tracking data were recorded using an EMR ACTUS infrared eye tracker (nac Image Technology Inc., Tokyo, Japan; sampling rate = 60 Hz; spatial resolution for eye movements < 0.5°). Following previous studies (Kennedy et al., 2017; Yamada and Kobayashi, 2018; Wang, 2019), binocular gaze trajectories were analyzed in raw form with minimal preprocessing of eye-tracking data. Specifically, the eye tracker outputs binocular gaze locations when both eyes were confidently identified, i.e., data were not recorded when either eye was undetectable, for example, due to blinking. Among these data, we excluded samples falling outside the image boundaries and where eye tracker estimation failed. We then included all remaining data of gaze locations in raw form for subsequent analyses. We excluded images from the analysis when gaze data were missing for more than 10% of the total viewing duration for the image. At the beginning of each recording session, we calibrated the eye tracker using a nine-point calibration and validation procedure, repeated up to three times if necessary.

### 2.4 Eye movement analysis

Characteristics of eye movements were quantified and compared through three types of measures: similarities in gaze allocations between participants, degree of visual exploration, and attention biases to specific image features. These eye movement features were calculated using the aforementioned binocular gaze location data. Mixed-model repeated-measures analyses of variance (ANOVAs) with Bonferroni *post-hoc* tests were performed to compare the effect of the diagnostic group on eye movement measurements, with the image as a random factor. We then further investigated the results through exploratory statistical and machine-learning analyses.

Similarities in spatial gaze allocations were assessed by correlating two gaze density maps (Kennedy et al., 2017). These maps were constructed by smoothing gaze locations using a fovea-sized 2D Gaussian kernel (i.e., 55 pixels in the 1600 × 1200 image) and were then normalized within each image. These gaze density maps represent the amount of gaze data looking at a particular location of the image. Each gaze density map was then flattened into a one-dimensional form, and the similarity between two gaze density maps was calculated using the Pearson correlation, which is thought to align closely with human intuition, despite its simplicity (Kennedy et al., 2017; Wang, 2019). This similarity metric was then used to assess whether each patient group had different gaze allocation compared with controls (CU participants). Specifically, the gaze similarity to controls was calculated for each participant and each image between the two groups, and then averaged for each of the three diagnostic groups. For the control group, we calculated the gaze similarity between all possible pairs within the control participants.

The degree of visual exploration was measured by the Shannon entropy of the gaze density map (Xu et al., 2014; Kennedy et al., 2017; Mikhailova et al., 2021). A higher entropy indicates a spatially diffused gaze to many different locations, while a lower entropy indicates a tightly focused gaze to only certain locations of the image. The Shannon entropy is defined as ∑_*i*_(−*p*_*i*_*log*_2_⁡*p*_*i*_), where *p*_*i*_indicates *i*-th pixel values of the gaze density map after dividing by the sum.

**FIGURE 2:**
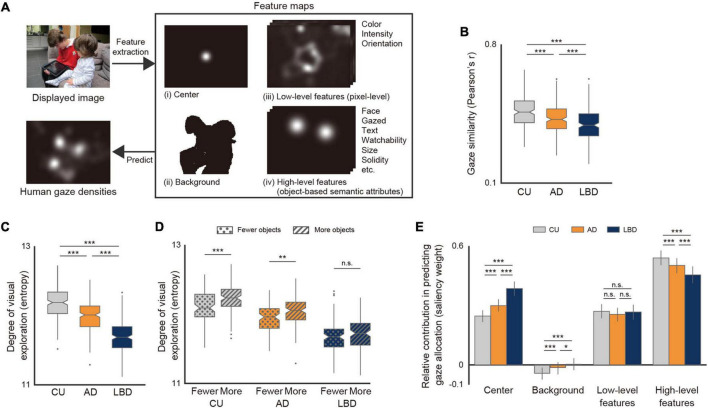
Altered patterns of spatial gaze allocations in the AD and LBD groups. **(A)** Overview of analysis using computational visual attention model. The images of visual stimuli were taken from the Object and Semantic Images and Eye-tracking dataset (Xu et al., 2014). **(B)** Similarity of gaze density maps with CU (one-way repeated-measures ANOVA with Bonferroni *post-hoc* test: *P* < 0.001). **(C)** Shannon entropy of gaze density maps (one-way repeated-measures ANOVA with Bonferroni *post-hoc* test: *P* < 0.001). **(D)** Shannon entropy of gaze density maps for images with fewer or more objects (two-way mixed-design ANOVA with Bonferroni *post-hoc* test, interaction: *P* = 0.021). **(E)** Relative contributions of four feature types to gaze allocations with 95% confidence intervals, derived from the analysis using the computational visual attention model (two-way repeated-measures ANOVA with Bonferroni *post-hoc* test, interaction: *P* < 0.001). Asterisks indicate significant differences between diagnostic groups: *P < 0.05, **P < 0.01, ***P < 0.001.

Attention bias to specific image features was evaluated using a computational visual attention model, namely a saliency model, to estimate the relative contribution of the following four feature types to gaze allocations (Xu et al., 2014; Wang et al., 2015): (i) image center, (ii) background (i.e., regions without labeled objects), (iii) low-level features (three pixel-level attributes), and (iv) high-level features (17 object-based semantic attributes). See Figure 2A for a schematic overview and the aforementioned subsection for the full list of attributes. The model has been described in detail in previous works (Xu et al., 2014; Wang et al., 2015). In summary, a binary classifier was trained to determine whether each location was looked at by linearly combining saliency values of each feature map extracted from the images. Feature weights resulting from the trained classifier were then used as the relative contribution of each feature type in predicting gaze allocation. The classifiers were trained for each diagnostic group, and the saliency weights between the groups were compared as group-level effects (Xu et al., 2014; Wang et al., 2015). For the ground-truth data (i.e., whether each location was looked at), positive samples were randomly selected from the pixels with the top 10% values in the gaze density maps, whereas negative samples were randomly selected from those with the lowest 30% values. The pixel-level feature maps were extracted using the most standard saliency model proposed by Itti et al. (1998), and the object- and semantic-level feature maps were calculated by placing a two-dimensional Gaussian kernel (σ = 2°) at each object’s center. The image-center map was calculated as a Gaussian map (σ = 1°) around the image center, and the background map was modeled as a binary map indicating regions without any labeled objects in the image. Before training the model, all samples extracted from each feature map were normalized to have a zero mean and unit variance. For the binary classifier, we used a support vector machine (SVM) classifier with a linear kernel (Boser et al., 1992) implemented with the scikit-learn package (version 0.23.2) in Python, leaving all parameters to their default values. All these parameters for the saliency-model-based analysis were the same as those in the previous study (Wang et al., 2015).

As an exploratory analysis regarding clinical-instrumental correlation, we investigated the associations of gaze allocation features with disease severity as well as with brain structural changes in AD and LBD patients. Disease severity measures included the Clinical Dementia Rating–Sum of Boxes (CDR-SB) score for dementia severity, MMSE score for assessing cognitive impairment, and gait speed for measuring motor impairments. Correlation and multiple linear regression analyses were used for disease severity and brain structural changes, respectively, after adjusting for age, sex, and years of education. For the multiple regression analysis, we applied a forward stepwise variable selection procedure based on the Bayesian information criterion to reduce the model complexity.

We then built machine learning-based classifiers to discriminate the three diagnostic groups on the basis of quantitative features characterizing gaze allocations. The features for the inputs were calculated for each participant and consisted of four feature types: the weights from the saliency model analysis; Shannon entropy; and proportion of gaze density and mean duration of gaze allocations on scene ROIs. To investigate whether the eye movement features could be used to differentiate AD and LBD patients at the individual level, the saliency models were trained for each participant, and four saliency weights for the image center, low- and high-level image features, and background were used. The Shannon entropy was calculated for each image, and its mean value across the images was used. The last two features related to the proportion of gaze density and mean duration of gaze allocations were calculated for each of the 15 scene ROIs consisting of objects with twelve types of semantic information; semantic objects (i.e., objects labeled with at least one type of semantic information); other objects (i.e., objects without any labeled semantic information); and background. Then, we used their means and standard deviations. For the classification algorithm, we used the same SVM classifier with a linear kernel and the default parameters. The model performance was evaluated with the area under the receiver operating characteristic curve (AUC), through a stratified 20-fold cross-validation procedure with 20 iterations with different training and test partitions. Three-class classifiers were built with a one-vs-one scheme and their AUC was computed as defined by Hand and Till (2001). To reduce model complexity and overfitting, feature selection was applied by using the least absolute shrinkage and selection operator penalized logistic regression model before the model training through the same 20-fold cross-validation procedure. Thus, the dataset was split into training (19/20) and test (1/20) partitions, where each training partition was used for the feature selection and model training. In total, 65 features of eye movements were used as inputs for the feature selection procedure, and selected features were fed into each classifier. The statistical significance of the classification performance was estimated by permutation testing. Specifically, we performed the aforementioned cross-validation procedure 100 times with randomly-permuted diagnosis labels to obtain a null distribution. We also compared performance against a baseline model incorporating clinical and demographic variables (i.e., the use of antipsychotic medication, MMSE score, age, sex, and years of education).

## 3 Results

### 3.1 Sample characteristics

Among the 142 participants who met the inclusion criteria, 36 participants were excluded from the present study (15, 17, and 4 participants from the CU, AD, and LBD groups, respectively) due to a calibration failure related to eye diseases (e.g., cataract) and failure to detect pupils caused by age-related reduction of pupil diameter and lax/redundant upper eyelid skin. Table 1 summarizes the characteristics of the participants included in the analysis (*N* = 106). Supplementary Tables 1–3 provide additional information related to clinical assessments and structural brain changes. The sample size of our dataset was comparable with those used in previous studies (Tseng et al., 2013; Wang et al., 2015).

The CU, AD, and LBD groups comprised 37, 49, and 20 participants, respectively, which satisfied the requirement of the power analysis. The AD and LBD groups included 31 and 13 MCI patients, respectively, and their proportions did not show any statistical difference (*P* = 0.892). For demographics, neither the age, sex, nor years of education were statistically different among the three diagnostic groups (all *P* > 0.05). None of the CU participants received antipsychotic medication, and the proportion of those on antipsychotic medication was the highest in the LBD group among the diagnostic groups (*P* = 0.001). All clinical, cognitive, and physical measures differed statistically among the groups except the Geriatric Depression Scale and Clock Drawing Test (see Table 1 for details).

### 3.2 Altered gaze allocations in AD and LBD patients

First, we directly investigated spatial gaze allocations by using gaze density maps aggregated for each diagnostic group. As qualitatively illustrated in Figure 1 (with additional examples in Supplementary Figure 1), compared with CU, AD and LBD patients focused on fewer locations (Figures 1A–D), exhibited different attention biases toward high-level semantic features (Figures 1E–H), and looked more at the image center (Figures 1I–L).

We then quantified the degree to which gaze allocations in AD and LBD patients differed from those in CU participants by calculating the similarity of the gaze density maps for each image. Consequently, the similarity in gaze allocations between CU participants and patients with AD or LBD was lower than that within CU participants (Figure 2B; repeated-measures ANOVA with Bonferroni *post-hoc* test: *P* < 0.001; AD vs. CU: *P* < 0.001, Hedges’ *g* = −0.43; LBD vs. CU: *P* < 0.001, Hedges’ *g* = −0.74), and the similarity was lower in LBD than in AD (*P* < 0.001, Hedges’ *g* = −0.34), indicating that AD and LBD patients exhibited different patterns of gaze allocations compared with CU participants, with the differences being more pronounced in LBD than in AD. These tendencies were consistently observed even at shorter timescales (Supplementary Figure 3A).

### 3.3 Diminished visual exploration

We investigated the degree of visual exploration by comparing the entropy of the gaze density maps. As hypothesized, LBD patients exhibited the lowest degree of entropy, followed by AD patients, and finally by CU participants (Figure 2C; repeated-measures ANOVA with Bonferroni *post-hoc* test: *P* < 0.001; LBD vs. AD: *P* < 0.001, Hedges’ *g* = −1.35; AD vs. CU: *P* < 0.001, Hedges’ *g* = −0.87). The result indicates that LBD and AD patients tended to focus on fewer locations in the scene and had a diminished visual exploration tendency compared with CU participants.

We further examined the associations between the degree of visual exploration and the characteristics of stimulus content, i.e., the number of objects. We first split the images into two groups with more and fewer objects by using their median value and then performed a two-way mixed-design ANOVA with a Bonferroni *post-hoc* test with the image group (more objects vs. fewer objects) as the between-subject factor and the diagnostic group as the within-subject factor. The result showed that AD patients and CU participants exhibited a greater degree of visual exploration in images with more objects (Figure 2D; interaction of diagnostic group × number of objects: *P* = 0.021; CU: *P* < 0.001, Hedges’ *g* = 0.61; AD: *P* = 0.010, Hedges’ *g* = 0.42), but LBD patients showed no statistical difference between images with more and fewer objects (*P* = 0.107, Hedges’ *g* = 0.23). This result suggests larger differences in the degree of visual exploration between the diagnostic groups with more objects in the scene. Note that the between-group differences measured by partial eta-squared increased with the number of objects in the scene (Supplementary Figure 3B; Pearson’s correlation: *r* = 0.17, *P* = 0.015), showing that the difference in the degree of visual exploration across the LBD, AD, and CU groups tended to be larger as the number of objects increased, partially because the degree of visual exploration in the LBD group was less affected by the number of objects than in the AD and CU groups.

We also examined whether the difference in visual exploration between the diagnostic groups could be related to that in participant fatigue or alertness. To this end, we examined changes in pupil diameter, a representative measure indicating increased fatigue (Yamada and Kobayashi, 2018), within the eye-tracking session and compared their differences between the diagnostic groups. Specifically, we split the images into the initial and latter halves of the session and then compared changes in pupil diameters using mixed-model repeated-measures ANOVA with the group (AD, LBD, and CU) and time (initial and latter halves) as independent factors. The result showed that neither the main effect of group (*P* = 0.916) nor the group × time interaction (*P* = 0.590) was statistically significant, suggesting that the diminished visual exploration in LBD and AD patients could not be attributed to the difference in participant fatigue or alertness.

### 3.4 Weaker attention bias to high-level image features

We next applied a computational visual attention model to evaluate the relative contribution of the four different factors in gaze allocation. Consequently, LBD patients, followed by AD patients, had a significantly weaker attention bias toward high-level image features, as well as greater image-center and background biases, whereas there was no statistical difference in the attention bias toward low-level image features of the pixel-level attributes (Figure 2E; two-way repeated-measures ANOVA with Bonferroni *post-hoc* test, interaction of diagnostic group × feature type: *P* < 0.001).

We conducted two additional analyses to further confirm the aforementioned results regarding reduced attention bias to high-level image features annotated on objects and increased center bias. First, we directly assessed gaze allocations to the objects in the scene. The results consistently aligned with those derived from the computational attention model analysis: AD and LBD patients showed a lower proportion of gaze allocations to the objects and a higher proportion to the background compared with CU participants (Supplementary Figure 4A; one-way repeated-measures ANOVA with Bonferroni *post-hoc* test: *P* < 0.001; AD vs. CU: *P* < 0.001, Hedges’ *g* = −0.16; LBD vs. CU: *P* < 0.001, Hedges’ *g* = −0.22). Additionally, there was an increase in the mean durations of gaze allocation to each object (Supplementary Figure 5A; *P* < 0.001; AD vs. CU: *P* = 0.045, Hedges’ *g* = 0.08; LBD vs. CU: *P* < 0.001, Hedges’ *g* = 0.43). In particular, the proportions of gaze allocations to text and human-made objects designed to be watched (i.e., watchability-related attributes e.g., a picture, display screen, and traffic sign) were lower in both AD and LBD patients (text: *P* < 0.001; AD vs. CU: *P* = 0.007, Hedges’ *g* = −0.12; LBD vs. CU: *P* < 0.001, Hedges’ *g* = −0.23; watchability: *P* < 0.001; AD vs. CU: *P* = 0.010, Hedges’ *g* = −0.07; LBD vs. CU: *P* < 0.001, Hedges’ *g* = −0.15; see Supplementary Figure 5B for full results). Second, we examined whether the tendency of AD and LBD patients to look at the image center could be explained by the distribution of objects in the images. Specifically, we investigated whether this center bias could still be observed in images with no objects in the center 2°circular area [the parameter was determined on the basis of the literature (Wang et al., 2015)]. Comparing the gaze density across the 34 images with no objects in the center, we found that the gaze density in the center was 1.3 times higher in the AD group and 2.6 times higher in the LBD group compared with the CU group (Supplementary Figure 4B; one-way repeated-measures ANOVA with Bonferroni *post-hoc* test: *P* < 0.001; AD vs. CU: *P* = 0.020, Hedges’ *g* = 0.38; LBD vs. CU: *P* < 0.001, Hedges’ *g* = 1.39).

Taken together, the results suggest that AD and LBD patients looked less at objects, along with a weaker attention bias toward high-level image features; while they looked more at the image center, even when there was no object present at the center.

As hypothesized, we confirmed that the LBD group, followed by the AD group, tended to have diminished visual exploration and a weaker attention bias toward high-level image features of object properties and semantic information. These tendencies persisted when the AD and LBD groups were subdivided in accordance with disease stage (i.e., MCI and dementia stages), with dementia stage-wise reductions in each patient group (Supplementary Figures 3C–E).

### 3.5 Associations of gaze allocation features with disease severity and brain structural changes

For the exploratory analyses regarding clinical-instrumental correlation, we further investigated the associations of altered patterns of visual exploration and attention biases with measures related to disease severity. These measures included the CDR-SB score for dementia severity, MMSE score for cognitive impairments, and gait speed for motor impairments. After adjusting for the covariates, correlation analyses revealed different associations within the AD and LBD groups.

First, the degrees of diminished visual exploration in the AD group correlated with lower MMSE scores (Spearman’s correlation: ρ = 0.34, *P* = 0.022), while those in the LBD group correlated mainly with slower gait speed (ρ = 0.70, *P* = 0.025). Note that neither MMSE scores in the LBD group nor gait speed in the AD group showed any statistical correlation with the degree of visual exploration (see Supplementary Table 4 for complete results). Second, the attention bias metrics derived from the computational attention model analysis also exhibited different association patterns between the diagnostic groups. In the AD group, a weaker attention bias toward high-level features correlated with CDR-SB, MMSE, and gait speed (CDR-SB: ρ = −0.33, *P* = 0.026; MMSE: ρ = 0.44, *P* = 0.002; gait speed: ρ = 0.33, *P* = 0.032), while the degree of center bias did not statistically correlate with any of the three measures investigated in this study (*P* > 0.05). In contrast, in the LBD group, the degree of center bias correlated with CDR-SB and MMSE scores (CDR-SB: ρ = 0.50, *P* = 0.042; MMSE: ρ = −0.53, *P* = 0.029), while the degree of attention bias toward high-level features showed no statistical correlations with any of the measures (*P* > 0.05). The correlation between the weaker attention bias and MMSE remained statistically significant even after correcting for multiple testing (Benjamini-Hochberg-corrected *P* = 0.036).

The different associations between eye movement patterns and disease severity measures between the AD and LBD groups suggest that these disease-specific alterations of eye movements may reflect distinct neuropathological changes in AD and LBD. Therefore, we conducted additional analyses on the associations with structural brain atrophies using stepwise multiple linear regression controlling for the covariates.

Consequently, in the AD group, the diminished visual exploration was associated with a reduced hippocampus volume (β = 2.3 × 10^2^, *P* = 0.037; Supplementary Table 5), and the reduced attention bias toward high-level features was associated with a thinning of the superior frontal cortex (β = 7.7 × 10^–1^, *P* = 0.005; Supplementary Table 5). In contrast, in the LBD group, neither the degree of visual exploration nor attention bias to high-level features was associated with structural changes in any of the ten brain ROIs, but the increased center bias was associated with the thinning of the superior parietal lobule (β = −1.1, *P* = 0.007; Supplementary Table 6).

These associations should be considered exploratory because of the small sample sizes and further validation is needed. However, these results suggest the following. (i) Diminished visual exploration may reflect cognitive impairment in AD patients and motor impairment in LBD patients, respectively. (ii) For the atypical bias in gaze allocations, the reduction in attention bias toward high-level image features may reflect disease severity in AD, while increased center bias may reflect severity in LBD. (iii) These alterations of eye movements in AD and LBD may reflect different patterns of neuropathological changes in each disease.

### 3.6 Classifier using eye movement features to identify/differentiate AD and LBD

**FIGURE 3:**
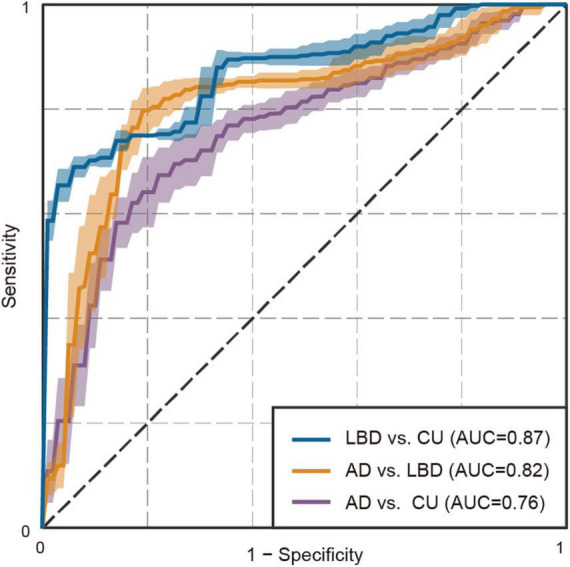
Receiver operating characteristic plot for the binary classifiers using eye movement features. Shades represent standard deviations.

Finally, we explored the extent to which these altered patterns of eye movements could differentiate AD and LBD patients. Through the cross-validation procedure, the models using features derived from eye movements achieved an AUC of 0.76 (95% confidence interval [CI]: 0.75 to 0.77, *P* < 0.01) for AD versus CU; an AUC of 0.87 (95% CI: 0.87 to 0.88, *P* < 0.01) for LBD versus CU; and an AUC of 0.82 (95% CI: 0.81 to 0.82, *P* < 0.01) for AD versus LBD (Figure 3; Supplementary Table 7 for full results). From the results of feature selection, each classification model mainly used fourteen eye movement features for AD versus CU; four features for LBD versus CU; and twelve features for AD versus LBD (Supplementary Table 8 for full results). The three-class AUC was 0.82 (95% CI: 0.81 to 0.82), which was much higher than the value of 0.59 (95% CI: 0.57 to 0.61) for the baseline model using clinical and demographic variables. When the performance was calculated for MCI stages, the models held reasonable performance, particularly for MCI-LB patients, with an AUC of 0.74 (95% CI: 0.73 to 0.75, *P* < 0.01) for MCI-AD versus CU; an AUC of 0.83 (95% CI: 0.82 to 0.83, *P* < 0.01) for MCI-LB versus CU; and an AUC of 0.75 (95% CI: 0.74 to 0.76, *P* < 0.01) for MCI-AD versus MCI-LB [3-class AUC of 0.77 (95% CI: 0.77 to 0.78), which was higher than the baseline model’s AUC of 0.56 (95% CI: 0.54 to 0.57)].

## 4 Discussion

The current study demonstrates two remarkable disease-specific altered patterns of gaze allocation to naturalistic complex scenes during free viewing in AD and LBD patients. First, both AD and LBD patients showed diminished visual exploration and tended to focus on fewer locations rather than exploring the entire image. AD patients tended to increase visual exploration in scenes with more objects in a similar way to CU participants, but the degree itself seems to be lower than CU regardless of the number of objects. In contrast, the degree of visual exploration in LBD patients was less affected by the number of objects in the scenes and held the lowest degree compared with CU participants and AD patients regardless of the number of objects. Furthermore, the degree of diminished visual exploration was differentially correlated with cognitive impairment in AD patients and motor impairments in LBD patients, respectively. Second, both AD and LBD patients exhibited a weaker attention bias toward high-level semantic features, looking less at objects, particularly texts and human-made objects designed to be watched. They also showed a stronger center bias even when there was no object at the center. These altered patterns of attention biases also showed different associations with dementia severity measures between the AD and LBD groups. Specifically, a reduction in the attention bias to high-level features was associated with dementia severity in the AD group, while an increase in the center bias was associated with dementia severity in the LBD group. Finally, these two distinct altered patterns in gaze allocations were consistently larger in LBD than in AD, enabling differentiation of the three diagnostic groups with an AUC of 0.82.

Our findings regarding diminished visual exploration are consistent with prior studies on AD patients (Daffner et al., 1992; Coco et al., 2021) and provide further support using a larger stimulus set that includes broader categories of objects. Additionally, we provide new insights into disease-specific associations between the degree of visual exploration and the number of objects in the scene, as well as cognitive/motor measures. According to the literature, diminished visual exploration could be attributed to at least three factors (Daffner et al., 1992, 1999; Anderson and MacAskill, 2013; Molitor et al., 2015; Seligman and Giovannetti, 2015): cognitive impairments (e.g., slowed processing speed and difficulties in disengaging and shifting attention), oculomotor dysfunctions (e.g., slowed velocity and increased latency of saccades), and apathy (e.g., a lack of motivation and interest). In the present study, the reduction of visual exploration in AD patients correlated with cognitive impairments and atrophy in the hippocampus, one of the regions associated with apathy (Johansson et al., 2020; Matuskova et al., 2021; Eggins et al., 2022), while that in LBD patients correlated with motor impairments. Our results indicate that the primary factors causing a reduction in visual exploration may differ between AD and LBD. Future studies that assess neuropsychiatric symptoms and oculomotor functions will be worthwhile to further elucidate this hypothesis.

Our results, derived from the computational attention model, demonstrate no statistical difference between diagnostic groups in the attention bias toward low-level features such as color and intensity. This suggests preserved bottom-up attentional guidance, which is consistent with prior studies (Landy et al., 2015; Shakespeare et al., 2015; Ramzaoui et al., 2018). Furthermore, our results newly show reduced attention bias toward high-level features of object-based semantic attributes, the extent of which correlates with dementia severity and the thinning of the superior frontal cortex in the AD group. Previous studies on AD patients reported impaired feature-conjunction search (Cormack et al., 2004; Landy et al., 2015) and reduced modulations of gaze allocations on the basis of task demands (Mosimann et al., 2004; Redel et al., 2012; Shakespeare et al., 2015; Tokushige et al., 2023), and they suggest impaired top-down attentional guidance in AD patients (Molitor et al., 2015; Ramzaoui et al., 2018). Moreover, high-level features in the scenes may affect top-down attentional control, along with numerous other factors including context and task-related factors (Mannan et al., 2009; Borji et al., 2012, 2013; Wang et al., 2015). The superior frontal cortex has also been implicated in top-down attentional control (Hopfinger et al., 2000; Baluch and Itti, 2011). Therefore, in alignment with previous studies, our findings may suggest impaired top-down attentional control in AD patients. Additionally, because high-level features related to object properties and semantic information play important roles in gaze allocations during specific tasks such as object search and memorization (Xu et al., 2014; Ramzaoui et al., 2018), our findings about the reduction in attention bias toward high-level features may provide another key difference for understanding deficits in activities of daily living observed in AD and LBD patients.

We observed that LBD patients looked more at the image center, even when there was no object, and the extent of this center bias was related to dementia severity. Although we believe no study in DLB exists, increased center bias has been reported in diseases with similar pathological changes (i.e., alpha-synucleinopathies) including Parkinson’s disease and multiple-system atrophy (Habibi et al., 2022). Furthermore, increased center bias has also been reported in multiple different neurological diseases such as autism spectrum disorder (Wang et al., 2015) and schizophrenia (Silberg et al., 2019), which we show in AD patients (although the extent was smaller than LBD). Since multiple, different neuropathological changes may affect the tendency to look at the image center, systematic comparisons across diseases will help further elucidate this complex association with neurological diseases.

In addition to greater alterations in LBD compared with AD, aligning with prior studies on eye movements using simple arrays (Cormack et al., 2004; Mosimann et al., 2005), we provide initial evidence for disease-specific alterations of eye movements to naturalistic complex scenes, suggesting that these altered patterns may reflect pathological changes of AD and LBD. This notion was corroborated by our results, including (i) the dementia stage-wise reductions in characteristics of altered gaze allocation patterns investigated in this study; and (ii) different associations of the altered eye movement patterns with structural brain atrophies in AD and LBD. Notably, with extensive knowledge of brain regions that control attention and gaze (Corbetta and Shulman, 2002; Anderson and MacAskill, 2013; Miller and Buschman, 2013; Leigh and Zee, 2015), altered patterns of eye movements could potentially identify particular brain regions affected by neurological disorders (Anderson and MacAskill, 2013; Itti, 2015; Terao et al., 2017). Compared with eye movements under structured tasks with simple artificial stimuli, spontaneous, voluntary eye movements can involve a wider range of brain regions, including higher cortical areas such as the temporal cortex, which is involved in processing high-level stimulus structures such as objects or gist, as well as the frontal cortex, which is involved in top-down attentional guidance (Miller and Buschman, 2013; Itti, 2015). From this perspective, our approach to multifacetedly characterize spontaneous gaze allocations to naturalistic complex scenes may provide an understanding of how changes in spontaneous gaze allocations relate to the pathophysiology of neurodegenerative dementias.

Our findings have at least two important clinical implications. First, eye movements not only reflect bottom-up and top-down attentional controls but also provide scaffolding for subsequent cognitive processing and influence activities in daily life. For example, eye movement patterns play an important functional role in a wide variety of daily activities, including making meals (Land et al., 1999; Hayhoe et al., 2003), reading the clock (Mosimann et al., 2004), understanding spoken language (Tanenhaus et al., 1995), identifying objects (Seligman and Giovannetti, 2015; Ramzaoui et al., 2018), and memorizing scenes (Coco et al., 2021) and faces (Henderson et al., 2005). In particular, recall performance for scenes and faces has been suggested to be influenced by the eye movement patterns during memory encoding (Olsen et al., 2016; Damiano and Walther, 2019; Coco et al., 2021), which include those reported as the altered patterns in AD and LBD patients in this study (i.e., diminished visual exploration, reduced fixations to objects, and increased center bias). Our findings of altered patterns of eye movements to complex, real-world scenes could offer a critical basis for understanding and mitigating the widespread impact of altered visual attention on activities in daily living. Examples may include studies on environmental modifications for enhancing attentional processing and improving daily-task performance, such as the provision of visually salient cues in object identification and wayfinding (Davis et al., 2017; Ramzaoui et al., 2022). Second, quantifying complex patterns of altered eye movements can support screening and differentiation for dementia subtypes, as has already been demonstrated for several other disorders (Anderson and MacAskill, 2013; Tseng et al., 2013). Note that behavioral measures collected using digital health technologies have become increasingly used in clinical trials of neurological disorders (Masanneck et al., 2023) and are expected to be used as non-invasive, relatively inexpensive, and easily measurable behavioral biomarkers (Kourtis et al., 2019; Schütz et al., 2022; Chen et al., 2023). In this context, along with other behavioral data such as speech, gait, and drawing (Mc Ardle et al., 2019; Pieruccini-Faria et al., 2021; Yamada et al., 2021, 2022a,b), eye-tracking data a prospective example (Anderson and MacAskill, 2013; Tseng et al., 2013; Itti, 2015; Kourtis et al., 2019; Valliappan et al., 2020). Additionally, compared with studies on eye movements under particular tasks (e.g., visual search, memory), the free-viewing paradigm used in this study has a methodological advantage in probing a wider range of individuals, including those with different languages or cognitive impairments that may affect the understanding of task instructions. Thus, it may provide a promising tool for a global approach to dementia research involving diverse populations (Gauthier et al., 2021; Dementia, 2023).

This study has several limitations. First, our dataset did not include neuropathological information regarding amyloid/tau pathology or postmortem follow-up. Because AD pathology often coincides with Lewy body pathology (Chatterjee et al., 2021), our samples may include patients with AD and Lewy body co-pathologies. Furthermore, motor impairments including bradykinesia have been recognized in non-parkinsonian neurodegenerative disorders including AD and have been proposed to signify the extent of widespread neurodegeneration regardless of associated pathology (Bologna et al., 2020; Paparella et al., 2021; Marsili et al., 2023). As eye movements and visual attention also require coordination between widespread neural circuits (Corbetta and Shulman, 2002; Anderson and MacAskill, 2013; Miller and Buschman, 2013; Leigh and Zee, 2015), the altered patterns of eye movements and their degrees may potentially be elucidated more directly from the perspective of network dysfunction of multiple brain regions affected by neurodegenerative diseases rather than the intrinsic neuropathology. Further studies with neuropathological assessments for AD, LBD, and co-pathology would enhance our comprehension of the neural mechanisms underlying altered patterns of eye movements reported in this study. Second, although we show altered patterns of eye movements in AD and LBD patients in a context closer to real-world viewing compared with prior studies using restricted stimuli, it raises an important further question to elucidate how these alterations affect eye movements during active participation in a real scene, rather than during passive observation like in this study. Future studies on eye movements during natural behaviors, such as activities of daily living (Land et al., 1999; Hayhoe et al., 2003; Davis et al., 2017), will further elucidate this issue and may help deepen our understanding of how altered eye movements affect everyday functioning. Third, we did not perform individual assessments of specific neuropsychological symptoms that could affect eye movements. For example, oculomotor abnormalities including saccadic hypometria and oculomotor apraxia have been identified in laboratory tests in various neurodegenerative dementias including AD and LBD (Boxer et al., 2012; Anderson and MacAskill, 2013), which could affect voluntary viewing behaviors as investigated in this study. In addition, although extensive attention dysfunctions, including sustained attention, that could influence oculomotor performance are known in DLB (Mosimann et al., 2005; Revie et al., 2019), we did not conduct attention-specific assessments such as the Stroop task. Further investigation may help explain the mechanisms of eye movement alterations in AD and LBD from neuropsychological viewpoints. Fourth, we assumed relatively large effect sizes for between-group differences on the basis of the literature and confirmed this assumption in our dataset. However, small but meaningful effects might be undetectable particularly in the exploratory analysis on clinical-instrumental correlations. Moreover, in the analysis, several correlations lost statistical significance after correction for multiple testing, even though we mainly reported the results without correction given the exploratory nature of the analysis. Our findings need to be validated with larger samples. Fifth, our main analysis combined MCI and dementia as one group due to small sample sizes, and disease stage-wise alterations were only investigated exploratorily. Further stage-wise analysis may reveal detailed patterns of altered eye movements at the prodromal stage, which would facilitate early dementia identification and differentiation. Sixth, although several confounding factors such as fatigue and antipsychotic medication were considered in the analysis, we did not adjust our analyses for non-antipsychotic medication and eye-related comorbidities that could also affect oculomotor functions and eye movement features. Seventh, our results on the classification models showed small CIs, but this may be due to the small sample size. Specifically, although we calculated the CIs through 20 repetitions of 20-fold cross validations, the small sample size made training and test sets largely overlapped across the repetitions, which might result in small CIs that did not properly represent the certainty of the results. To show generalizability of the classification models using eye movement features, we need to validate with larger samples and those collected in independent, different institutions.

In this study on free viewing of naturalistic complex scenes, we show distinct altered patterns of eye movements associated with disease severity in AD and LBD patients at the prodromal and dementia stages: (i) diminished visual exploration, differentially associated with cognitive impairment in AD and with motor impairment in LBD; and (ii) reduced gaze allocations to objects attributed to a weaker attention bias toward object-based semantic attributes in AD and attributed to a greater center bias in LBD. These distinct altered patterns may help differentiate AD and LBD as behavioral markers and deepen our understanding of how they manifest in the real world, ultimately leading to the mitigation of the widespread impact of impaired visual attention on activities of daily living.

## Data availability statement

The datasets presented in this article are not readily available because they contain information that could compromise research participant privacy/consent. Anonymized data that support the findings of this study may be made available upon reasonable request for academic purposes. Requests to access the datasets should be directed to YY, ysnr@jp.ibm.com.

## Ethics statement

The studies involving humans were approved by the Ethics Committee, University of Tsukuba Hospital (H29-065). The studies were conducted in accordance with the local legislation and institutional requirements. The participants provided their written informed consent to participate in this study.

## Author contributions

YY: Conceptualization, Formal analysis, Investigation, Methodology, Visualization, Writing – original draft, Writing – review & editing. KS: Investigation, Visualization, Writing – review & editing. MK: Formal analysis, Writing – review & editing. MN: Data curation, Funding acquisition, Investigation, Methodology, Writing – review & editing. MO: Investigation, Writing – review & editing. KN: Formal analysis, Funding acquisition, Investigation, Methodology, Writing – review & editing. TA: Funding acquisition, Investigation, Methodology, Project administration, Writing – review & editing.
