# Supplementary material for: Distinct eye movement patterns to complex scenes in Alzheimer’s disease and Lewy body disease
Source: Front Neurosci. 2024 Apr 5;18:1333894. doi: 10.3389/fnins.2024.1333894 (PMC11026598; doi:10.3389/fnins.2024.1333894)
Supplement: Supplementary file 1 [file Data_Sheet_1.docx]

Supplementary Material for Distinct eye movement patterns to complex scenes in Alzheimer's disease and Lewy body disease

# Supplementary Figures and Tables

## Supplementary Figures


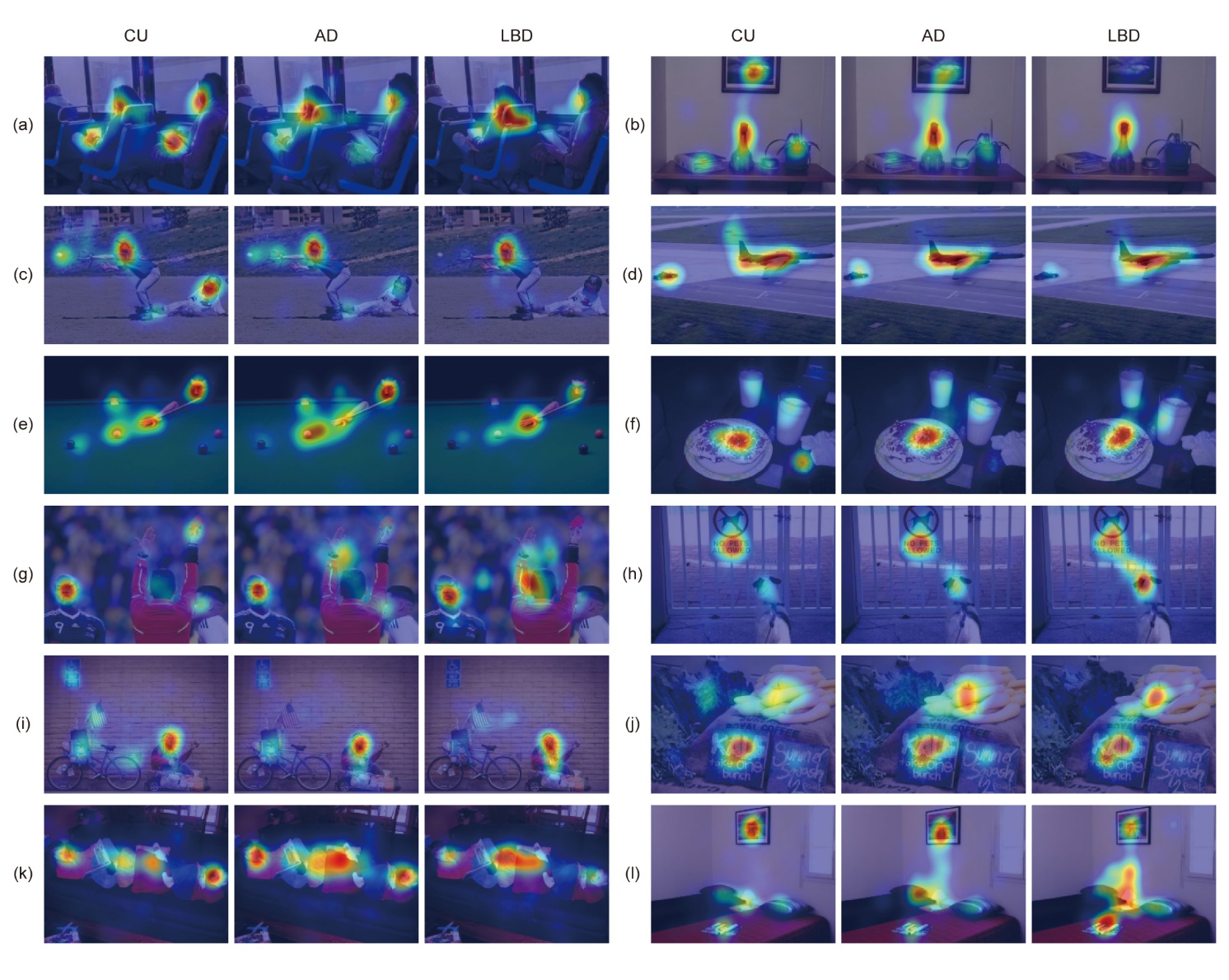


**Supplementary Figure S1.** Other examples of complex scene images with gaze densities derived from the CU, AD, and LBD groups. Results show that AD and LBD patients focused on fewer locations (a–f), had different attention biases toward high-level image features of object-based sematic attributes (g–j), and looked more at the image center (k, l), compared with CU participants. The images of visual stimuli were taken from the Object and Semantic Images and Eye-tracking dataset (Xu et al., 2014).

**
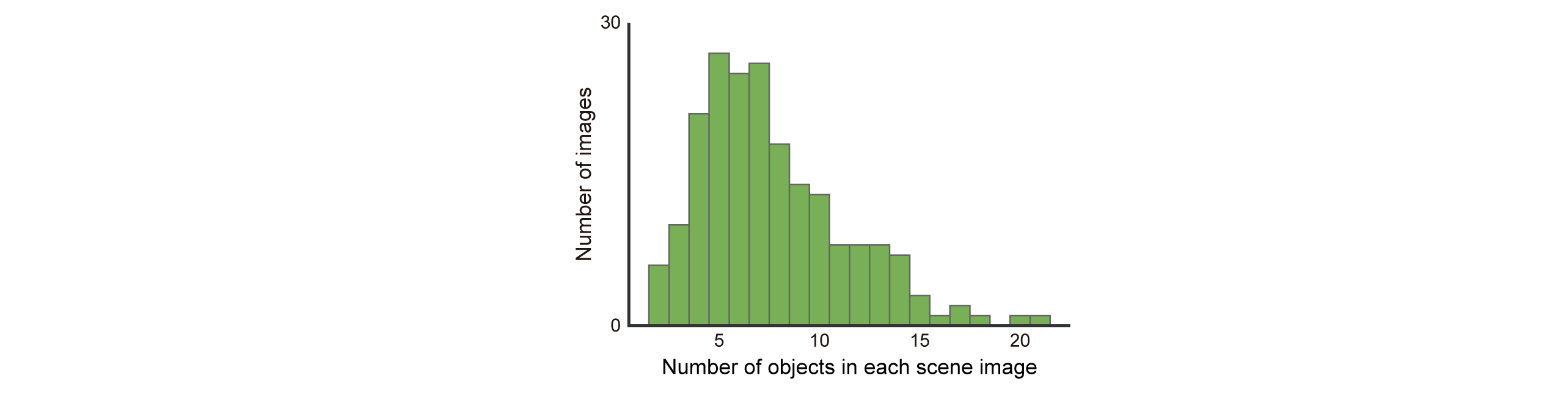
**

**Supplementary Figure S2.** Histogram of numbers of objects in each scene image.


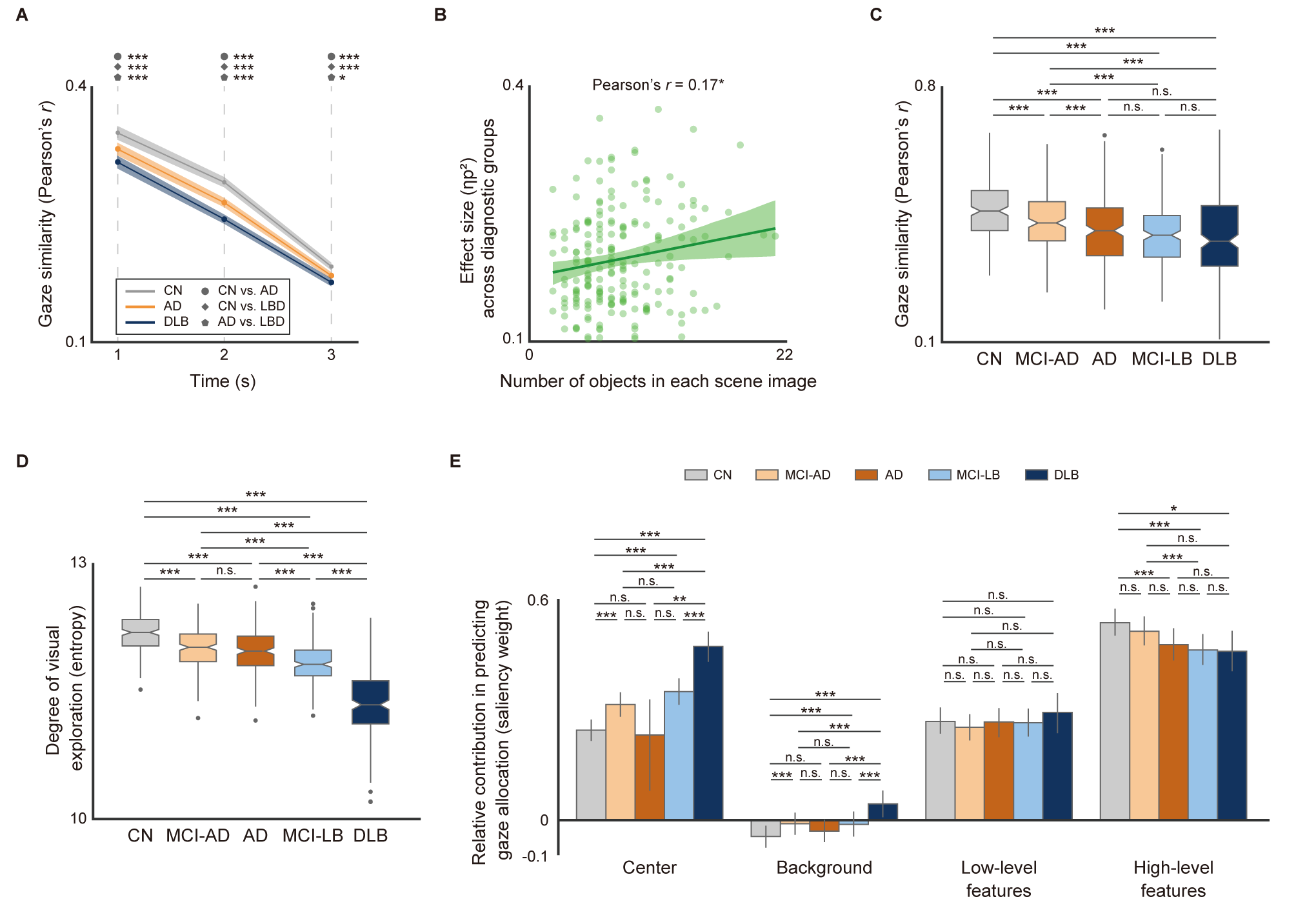


**Supplementary Figure S3.** Altered patterns of spatial gaze allocations in AD and LBD patients. **(A)** Temporal evolution of similarity of the gaze density maps with CU (one-way repeated-measures ANOVA with Bonferroni post-hoc test for each time bin: *P* < 0.001 for all). Shades represent standard errors of the mean. **(B)** Associations of differences in the degree of visual exploration in terms of effect size across the AD, LBD, and CU groups with numbers of objects in each scene image (Pearson's correlation: *r* = 0.17, *P* = 0.015). **(C)** Similarity of the gaze density maps with CU (one-way repeated-measures ANOVA with Bonferroni post-hoc test: *P* < 0.001). **(D)** Shannon entropy of gaze density maps (one-way repeated-measures ANOVA with Bonferroni post-hoc test: *P* < 0.001). **(E)** Relative contributions of four feature types to gaze allocations with 95% confidence intervals, derived from the analysis using the computational visual attention model (two-way repeated-measures ANOVA, interaction of diagnostic group × feature type: *P* < 0.001). Asterisks indicate significant differences between diagnostic groups: **P* < 0.05, ***P* < 0.01, ****P* < 0.001.


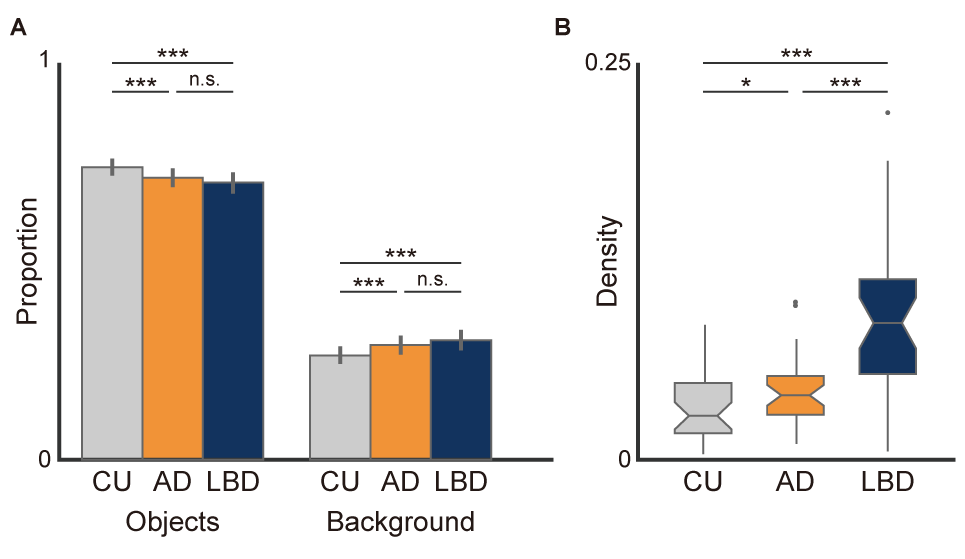


**Supplementary Figure S4.** Corroborating results for weaker attention bias toward object-based semantic attributes and greater center bias in AD and LBD. **(A)** Proportion of gaze allocations to objects or to the background with 95% confidence intervals (one-way repeated-measures ANOVA with Bonferroni post-hoc test: *P* < 0.001 for both). **(B)** Gaze density across images with no objects in the center (one-way repeated-measures ANOVA with Bonferroni post-hoc test: *P* < 0.001). Asterisks indicate significant differences between diagnostic groups: **P* < 0.05, ***P* < 0.01, ****P* < 0.001.


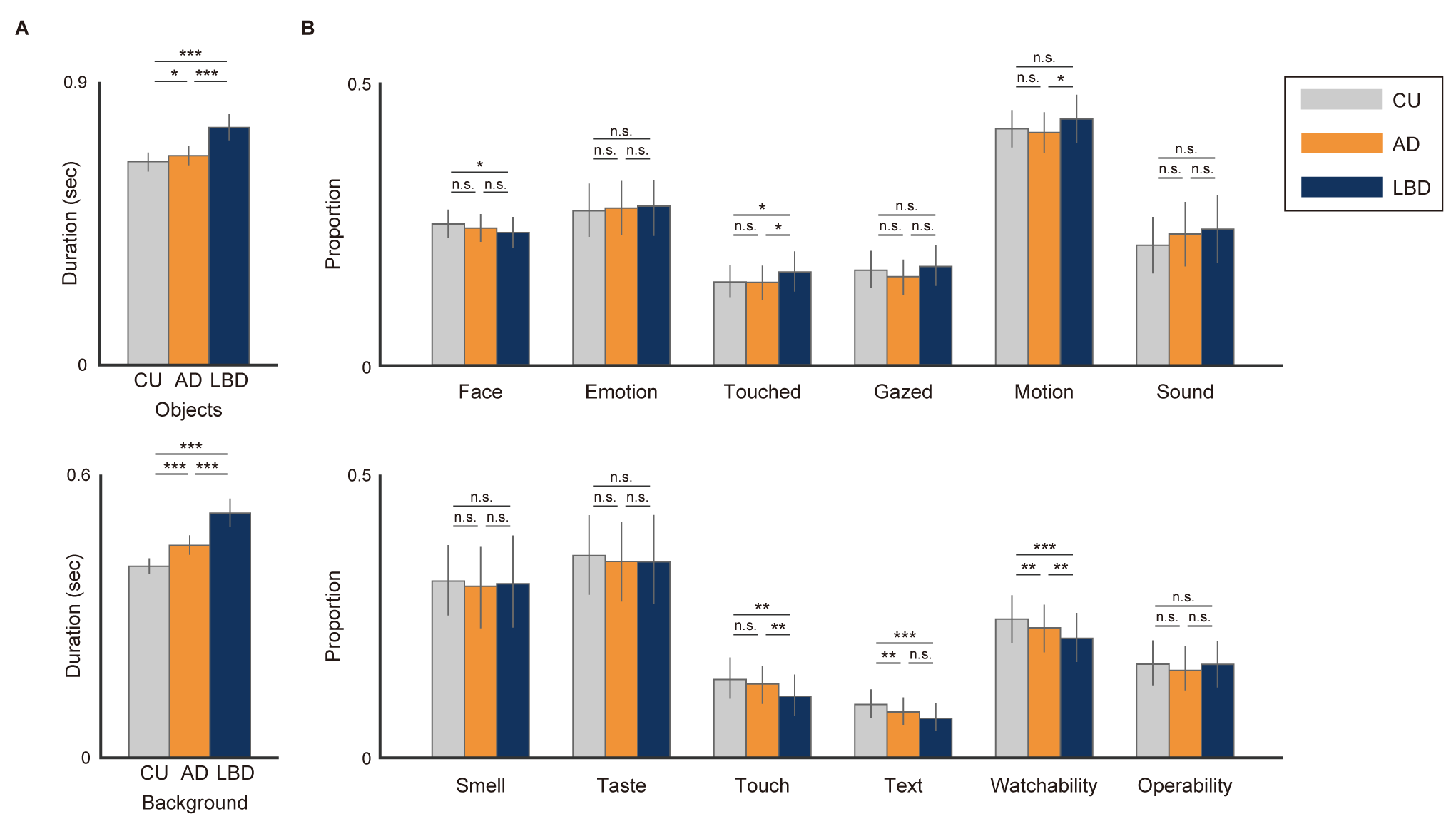


**Supplementary Figure S5.** Mean duration and proportion of gaze allocations. **(A)** Mean durations of gaze allocations to objects and the background (one-way repeated-measures ANOVA with Bonferroni post-hoc test, objects: *P* < 0.001; background: *P* < 0.001). **(B)** Proportion of gaze allocations to each object-based semantic attribute (one-way repeated-measures ANOVA with Bonferroni post-hoc test: *P* < 0.001 to 0.811). Error bars denote 95% confidence intervals. Asterisks indicate significant differences between diagnostic groups: **P* < 0.05, ***P* < 0.01, ****P* < 0.001.

## Supplementary Tables

**Supplementary Table S1.** Clinical information for the LBD group.

|  | **MCI-LB (*n*=13)** | | **DLB (*n*=7)** | |
| --- | --- | --- | --- | --- |
| Core features, *n* (%) |  |  |  |  |
| Cognitive fluctuations | 2 | (15.4%) | 6 | (85.7%) |
| Visual hallucinations | 2 | (15.4%) | 3 | (42.9%) |
| REM sleep behavior disorder | 9 | (69.2%) | 4 | (57.1%) |
| Parkinsonism | 5 | (38.5%) | 3 | (42.9%) |
| Five-item Unified Parkinson’s Disease Rating Scale, mean (SD) | 2.2 | (1.5) | 2.9 | (2.8) |

Abbreviations: LBD, Lewy body disease; DLB, dementia with Lewy bodies; MCI-LB, mild cognitive impairment with Lewy bodies; REM, rapid eye movement; SD, standard deviation.

**Supplementary Table S2.** Dementia stage-wise structural brain atrophies in the CU and AD groups.

|  |  |  |  |  |  |  | ***P* value** | |
| --- | --- | --- | --- | --- | --- | --- | --- | --- |
|  | **CU (*n*=37)** | | **MCI-AD (*n*=31)** | | **AD (*n*=18)** | | Unadjusted | Adjusted |
| Subcortical volume, normalized to intracranial volume, ×10^-3^ | | | | | | | | |
| Hippocampus | 2.48 | (0.24) | 2.25 | (0.36) | 2.01 | (0.44) | **<0.001** | **<0.001** |
| Cortical thickness, mm |  |  |  |  |  |  |  |  |
| Middle temporal | 2.64 | (0.12) | 2.56 | (0.15) | 2.44 | (0.17) | **<0.001** | **<0.001** |
| Inferior temporal | 2.73 | (0.11) | 2.67 | (0.15) | 2.57 | (0.18) | **0.001** | **0.011** |
| Inferior parietal | 2.27 | (0.11) | 2.20 | (0.13) | 2.11 | (0.13) | **<0.001** | **<0.001** |
| Superior parietal | 2.05 | (0.13) | 2.01 | (0.14) | 1.96 | (0.15) | 0.075 | **0.013** |
| Supra marginal | 2.37 | (0.13) | 2.29 | (0.13) | 2.21 | (0.12) | **<0.001** | **<0.001** |
| Superior frontal | 2.58 | (0.11) | 2.55 | (0.14) | 2.45 | (0.11) | **<0.001** | **0.002** |
| Entorhinal | 3.40 | (0.25) | 3.08 | (0.39) | 2.68 | (0.56) | **<0.001** | **<0.001** |
| Fusiform | 2.55 | (0.13) | 2.49 | (0.12) | 2.38 | (0.15) | **<0.001** | **0.003** |
| Precuneus | 2.21 | (0.13) | 2.17 | (0.11) | 2.07 | (0.10) | **<0.001** | **0.002** |

Values are displayed as mean (SD) and were examined by using one-way analysis of variance adjusted and unadjusted for age, sex, and years of education, with Benjamini-Hochberg correction for multiple testing. Bold values highlight statistically significant differences.

Abbreviations: AD, Alzheimer's disease; MCI-AD, mild cognitive impairment due to AD; CU, cognitively unimpaired; SD, standard deviation.

**Supplementary Table S3.** Dementia stage-wise structural brain atrophies in the CU and LBD groups.

|  |  |  |  |  |  |  | ***P* value** | |
| --- | --- | --- | --- | --- | --- | --- | --- | --- |
|  | **CU (*n*=37)** | | **MCI-LB (*n*=13)** | | **DLB (*n*=7)** | | Unadjusted | Adjusted |
| Subcortical volume, normalized to intracranial volume, ×10^-3^ | | | | | | | | |
| Hippocampus | 2.48 | (0.24) | 2.36 | (0.25) | 2.27 | (0.28) | 0.081 | 0.202 |
| Cortical thickness, mm |  |  |  |  |  |  |  |  |
| Middle temporal | 2.64 | (0.12) | 2.61 | (0.11) | 2.46 | (0.15) | **0.024** | **0.035** |
| Inferior temporal | 2.73 | (0.11) | 2.73 | (0.10) | 2.59 | (0.16) | **0.044** | 0.081 |
| Inferior parietal | 2.27 | (0.11) | 2.29 | (0.13) | 2.09 | (0.16) | **0.018** | **0.035** |
| Superior parietal | 2.05 | (0.13) | 2.08 | (0.13) | 1.93 | (0.12) | **0.050** | 0.108 |
| Supra marginal | 2.37 | (0.13) | 2.36 | (0.11) | 2.23 | (0.10) | **0.044** | 0.075 |
| Superior frontal | 2.58 | (0.11) | 2.57 | (0.11) | 2.45 | (0.16) | **0.044** | 0.112 |
| Entorhinal | 3.40 | (0.25) | 3.23 | (0.39) | 3.09 | (0.22) | **0.044** | 0.112 |
| Fusiform | 2.55 | (0.13) | 2.50 | (0.10) | 2.42 | (0.08) | **0.046** | 0.112 |
| Precuneus | 2.21 | (0.13) | 2.20 | (0.11) | 2.08 | (0.13) | **0.046** | 0.112 |

Values are displayed as mean (SD) and were examined by using one-way analysis of variance adjusted and unadjusted for age, sex, and years of education, with Benjamini-Hochberg correction for multiple testing. Bold values highlight statistically significant differences.

Abbreviations: LBD, Lewy body disease; DLB, dementia with Lewy bodies; MCI-LB, mild cognitive impairment with Lewy bodies; CU, cognitively unimpaired; SD, standard deviation.

**Supplementary Table S4.** Association of gaze allocation features with dementia severity (CDR-SB), cognitive impairments (MMSE), and motor impairments (gait speed) via Spearman's correlation analyses adjusted for age, sex, and years of education.

|  | **CDR-SB** | |  | **MMSE** | |  | **Gait speed** | |
| --- | --- | --- | --- | --- | --- | --- | --- | --- |
|  | *ρ* | *P* value (Corrected) |  | *ρ* | *P* value (Corrected) |  | *ρ* | *P* value (Corrected) |
| Visual exploration | | | | | | | | |
| AD group | −0.246 | 0.100 (0.200) |  | **0.336** | **0.022** (0.095) |  | 0.139 | 0.380 (0.489) |
| LBD group | **−0.505** | **0.039** (0.095) |  | 0.295 | 0.251 (0.402) |  | **0.698** | **0.025** (0.095) |
| Center bias | | | | | | | | |
| AD group | 0.002 | 0.987 (0.987) |  | −0.114 | 0.451 (0.537) |  | −0.167 | 0.290 (0.402) |
| LBD group | **0.497** | **0.042** (0.095) |  | **−0.528** | **0.029** (0.095) |  | −0.380 | 0.279 (0.402) |
| Attention bias to high-level image features | | | | | | | | |
| AD group | **−0.328** | **0.026** (0.095) |  | **0.440** | **0.002 (0.036)** |  | **0.332** | **0.032** (0.095) |
| LBD group | −0.315 | 0.219 (0.394) |  | 0.185 | 0.477 (0.537) |  | 0.045 | 0.901 (0.954) |

Values in parentheses show Benjamini-Hochberg-corrected *P* values for multiple testing. Bold values highlight statistically significant correlations.

Abbreviations: CDR-SB, Clinical Dementia Rating–Sum of Boxes; MMSE, Mini-Mental State Examination; AD, Alzheimer's disease; LBD, Lewy body disease.

**Supplementary Table S5.** Associations of the alterations in eye movements with structural brain atrophies in the AD group via stepwise linear regression analyses adjusted for age, sex, and years of education.

|  | **Visual exploration** | | |  | **Attention bias to  high-level image features** | | |  | **Center bias** | | |
| --- | --- | --- | --- | --- | --- | --- | --- | --- | --- | --- | --- |
|  | *β* | (SE) | *P* value |  | *β* | (SE) | *P* value |  | *β* | (SE) | *P* value |
| Subcortical volume, normalized to intracranial volume, ×10^−3^ | | | | | | | | | | | |
| Hippocampus | **0.231** | **(0.108)** | **0.037** |  | – | | – |  | – | | – |
| Cortical thickness, mm | | | | | | | | | | | |
| Middle temporal | – | | – |  | – | | – |  | – | | – |
| Inferior temporal | – | | – |  | – | | – |  | – | | – |
| Inferior parietal | – | | – |  | – | | – |  | – | | – |
| Superior parietal | – | | – |  | – | | – |  | – | | – |
| Supra marginal | – | | – |  | – | | – |  | – | | – |
| Superior frontal | – | | – |  | **0.767** | **(0.262)** | **0.005** |  | – | | – |
| Entorhinal | – | | – |  | – | | – |  | – | | – |
| Fusiform | – | | – |  | −0.528 | (0.265) | 0.053 |  | – | | – |
| Precuneus | – | | – |  | – | | – |  | – | | – |

Bold values highlight statistically significant associations.

Abbreviations: AD, Alzheimer's disease

**Supplementary Table S6.** Associations of the alterations in eye movements with structural brain atrophies in the LBD group via stepwise linear regression analyses adjusted for age, sex, and years of education.

|  | **Visual exploration** | | |  | **Attention bias to  high-level image features** | | |  | **Center bias** | | |
| --- | --- | --- | --- | --- | --- | --- | --- | --- | --- | --- | --- |
|  | *β* | (SE) | *P* value |  | *β* | (SE) | *P* value |  | *β* | (SE) | *P* value |
| Subcortical volume, normalized to intracranial volume, ×10^−3^ | | | | | | | | | | | |
| Hippocampus | – | | – |  | – | | – |  | – | | – |
| Cortical thickness, mm | | | | | | | | | | | |
| Middle temporal | – | | – |  | – | | – |  | – | | – |
| Inferior temporal | – | | – |  | – | | – |  | – | | – |
| Inferior parietal | – | | – |  | – | | – |  | – | | – |
| Superior parietal | – | | – |  | – | | – |  | **−1.086** | **(0.343)** | **0.007** |
| Supra marginal | – | | – |  | – | | – |  | – | | – |
| Superior frontal | – | | – |  | – | | – |  | 0.685 | (0.376) | 0.090 |
| Entorhinal | – | | – |  | – | | – |  | – | | – |
| Fusiform | – | | – |  | – | | – |  | – | | – |
| Precuneus | – | | – |  | – | | – |  | – | | – |

Bold values highlight statistically significant associations.

Abbreviations: LBD, Lewy body disease.

**Supplementary Table S7.** Performance of classification model using gaze allocation features. The model performance was evaluated with a 20-fold cross-validation procedure.

|  | Mean [95% Confidence Interval] | | | | | | | | | |
| --- | --- | --- | --- | --- | --- | --- | --- | --- | --- | --- |
|  | **AUC** | | **Accuracy (%)** | | **Sensitivity (%)** | | **Specificity (%)** | | **F1-score (%)** | |
| **AD vs. CU** | 0.762 | [0.754, 0.770] | 71.7 | [70.5, 72.9] | 71.9 | [70.2, 73.7] | 71.4 | [70.2, 73.7] | 74.3 | [73.1, 75.5] |
| **LBD vs. CU** | 0.873 | [0.870, 0.876] | 85.0 | [84.2, 85.8] | 66.0 | [65.0, 67.0] | 95.3 | [94.3, 96.3] | 75.6 | [74.5, 76.7] |
| **AD vs. LBD** | 0.816 | [0.810, 0.822] | 80.9 | [80.4, 81.5] | 64.8 | [64.2, 65.3] | 87.6 | [86.9, 88.2] | 66.3 | [65.6, 67.1] |

Abbreviations: AUC, area under the receiver operating characteristic curve; CU, cognitively unimpaired; AD, Alzheimer's disease; LBD, Lewy body disease.

**Supplementary Table S8.** Eye movement features robustly selected across different training sets for each binary classifier.

| **Classifier** | **Selected features** |
| --- | --- |
| **AD vs. CU** (13 features) | Saliency weight for the image center.  Mean of gaze density proportion to the ROIs related to sound and taste.  SD of gaze density proportion to the ROIs related to semantic objects, touch, sound, and taste.  Mean of gaze allocation duration to the ROIs related to sound and smell.  SD of gaze allocation duration to the ROIs related to watchability, text, touched, and sound. |
| **LBD vs. CU** (4 features) | Mean of gaze density proportion to the ROIs related to taste.  SD of gaze density proportion to the ROIs related to semantic objects and smell.  Mean of gaze allocation duration to the ROIs related to gazed. |
| **AD vs. LBD** (12 features) | Mean of gaze density proportion to the ROIs related to semantic objects, gazed, touch, touched, and other objects.  SD of gaze density proportion to the ROIs related to sound, smell, and taste.  Mean of gaze allocation duration to the ROIs related to gazed, touched, emotion, and touch. |

Abbreviations: CU, cognitively unimpaired; AD, Alzheimer's disease; LBD, Lewy body disease; SD, standard deviation; ROI, region of interest.
